# The Expression of VEGF-A Is Down Regulated in Peripheral Blood Mononuclear Cells of Patients with Secondary Progressive Multiple Sclerosis

**DOI:** 10.1371/journal.pone.0019138

**Published:** 2011-05-06

**Authors:** Ellen Iacobaeus, Petra Amoudruz, Mikael Ström, Mohsen Khademi, Lou Brundin, Jan Hillert, Ingrid Kockum, Vivianne Malmström, Tomas Olsson, Emma Tham, Fredrik Piehl

**Affiliations:** 1 Department of Clinical Neuroscience, Neuroimmunology Unit, Karolinska Institute Solna, Center for Molecular Medicine, Stockholm, Sweden; 2 Department of Medicine, Rheumatology Unit, Karolinska Institute Solna, Center for Molecular Medicine, Stockholm, Sweden; 3 Department of Clinical Neuroscience, The Multiple Sclerosis Research Group, Karolinska Institute Solna, Center for Molecular Medicine, Stockholm, Sweden; 4 Department of Clinical Genetics, L5:03, Karolinska University Hospital, Solna, Sweden; Boston University School of Medicine, United States of America

## Abstract

**Background:**

Most patients with relapsing-remitting multiple sclerosis (RRMS) eventually enter a secondary progressive (SPMS) phase, characterized by increasing neurological disability. The mechanisms underlying transition to SPMS are unknown and effective treatments and biomarkers are lacking. Vascular endothelial growth factor-A (VEGF-A) is an angiogenic factor with neuroprotective effects that has been associated with neurodegenerative diseases. SPMS has a prominent neurodegenerative facet and we investigated a possible role for VEGF-A during transition from RRMS to SPMS.

**Methodology/Principal Findings:**

VEGF-A mRNA expression in peripheral blood mononuclear (PBMC) and cerebrospinal fluid (CSF) cells from RRMS (n = 128), SPMS (n = 55) and controls (n = 116) were analyzed using real time PCR. We demonstrate reduced expression of VEGF-A mRNA in MS CSF cells compared to controls (*p*<0.001) irrespective of disease course and expression levels are restored by natalizumab treatment(*p*<0.001). VEGF-A was primarily expressed in monocytes and our CSF findings in part may be explained by effects on relative monocyte proportions. However, VEGF-A mRNA expression was also down regulated in the peripheral compartment of SPMS (*p*<0.001), despite unchanged monocyte counts, demonstrating a particular phenotype differentiating SPMS from RRMS and controls. A possible association of allelic variability in the *VEGF-A* gene to risk of MS was also studied by genotyping for six single nucleotide polymorphisms (SNPs) in MS (n = 1114) and controls (n = 1234), which, however, did not demonstrate any significant association between VEGF-A alleles and risk of MS.

**Conclusions/Significance:**

Expression of VEGF-A in CSF cells is reduced in MS patients compared to controls irrespective of disease course. In addition, SPMS patients display reduced VEGF-A mRNA expression in PBMC, which distinguish them from RRMS and controls. This indicates a possible role for VEGF-A in the mechanisms regulating transition to SPMS. Decreased levels of PBMC VEGF-A mRNA expression should be further evaluated as a biomarker for SPMS.

## Introduction

Multiple Sclerosis (MS) is a chronic inflammatory demyelinating disease of the central nervous system (CNS). A majority of patients initially display a relapsing-remitting disease course (RRMS), characterized by inflammatory demyelinating attacks, axonal injury and varying degree of repair [1]. However, most RRMS patients with time enter a secondary progressive phase (SPMS), with failure of repair mechanisms leading to gradual neurodegeneration and a continuous accumulation of disability [1]. The strongest predictive factors favoring an earlier transition to a progressive disease course are efferent symptoms and lack of complete remission of the onset bout [2].The neurodegenerative component in progressive MS is more prominent and disease modulatory treatments, even those shown to be effective in breakthrough RRMS disease, have only limited effects in SPMS [3], [4], [5].Very little is known about the mechanisms underlying the transition to progressive MS disease and the diagnosis of a progressive disease course is made solely on clinical grounds, since reliable supporting laboratory or neuroradiological tests are lacking [6]. In clinical practice it is often difficult to determine when the progressive disease starts until it has already been going on for a longer time, which makes it complicated to use as an outcome in clinical trials and also poses a clinical problem for treatment decisions.

A number of potential biomarkers for monitoring of MS disease course, response to therapies and prediction of disability have been studied, some of them with promising results [7], [8]. However, there is a scarcity of candidates evaluated as biomarkers for SPMS, although a recent study demonstrated that levels of complement factor H is elevated in SPMS compared to RRMS [9]. Vascular endothelial growth factor A (VEGF-A) is an angiogenic and pro-inflammatory factor with neuroprotective effects on neuronal and glial cells that also stimulates proliferation and survival of neural stem cells [10], [11]. Studies on the neurodegenerative diseases amyotrophic lateral sclerosis (ALS) and Alzheimer's disease (AD) have suggested a role for VEGF-A in the pathogenesis of these diseases and a genetic association between *VEGF-A* variants and these diseases has been demonstrated [12], [13], [14], [15], [16], [17], [18]. Results from a few smaller studies have also indicated a role for VEGF-A in MS [19], [20], [21], [22], [23]. We previously observed decreased levels of VEGF-A in cerebrospinal fluid (CSF) cells from a small group of MS patients compared to controls [22].The objective of this study was to determine VEGF-A expression profiles in CSF and peripheral blood in a large cohort of well characterized MS patients and relate this to different disease measures. In addition, we have investigated if genetic variability in the *VEGF-A* gene is associated to risk of MS.

## Methods

### Ethics Statement

The study was approved by the regional ethical committee, “Regionala etikprövningsnämnden i Stockholm, EPN” and written informed consent was obtained from all patients.

### Patients

All CSF- and peripheral blood mononuclear cells (PBMC) samples were obtained from our in-house biobank containing samples collected, during routine neurological diagnostic work up or during clinical follow-up, from 2001 to 2009. Samples from natalizumab treated patients were collected before and after one year of treatment for study purposes. Demographic data of the patients included in the expression studies are described in Table S1. Two patient cohorts were used for VEGF-A expression analysis; *study group A* composed of RRMS (n = 63), SPMS (n = 35) and controls with other non-inflammatory neurological diseases (OND) (n = 68), and *study group B* used for replication with RRMS (n = 65), SPMS (n = 20) and OND (n = 48). All MS patients fulfilled the McDonald criteria [24]. Classification and scoring of MS patients was assessed by a trained neurologist. For RRMS, a relapse was defined as an increase with ≥1 point on the expanded disability status scale (EDSS), with duration of at least one week before sampling, where systemic infection had been ruled out. Remission was defined as a stable clinical status >3 months prior to sampling. SPMS was defined as an initial relapsing-remitting disease course followed by more than 12 months of continuous worsening of neurological function (≥0.5 EDSS point) not explained by relapses. At time of sampling, 14 RRMS patients were on immunomodulatory treatment including IFN-β1a (n = 8), IFN-β1b (n = 3), glatiramer acetate (n = 2) and intravenous immunoglobulins (n = 1), and 2 OND patients were on low doses of oral corticosteroids. Magnetic resonance imaging (MRI) data obtained within 2 months of sampling was available in 80 cases. Routine MRI examinations and determination of oligoclonal bands and IgG index in CSF and serum were performed as described previously [25]. The OND control groups included patients with diagnoses listed in Table S2.

### Sample preparation and mRNA expression analysis

CSF cells were collected and processed as previously described [26]. Peripheral blood was collected in sodium citrate-containing cell preparation tubes (Vacutainer CPT; BD Biosciences, San Jose, California, USA) and EDTA tubes. PBMC were separated by density gradient centrifugation and cells from the interphase were collected and washed twice with Dulbeccos's PBS. Cell pellets were frozen on dry ice and stored at −80°C until analysis. Total RNA was extracted and reverse transcribed as previously described [26]. Two RT-PCR expression assays were used; The SYBR-Green-based protocol was used for analysis on study group A and the TaqMan gene expression assay was used for study group B. A BioRad iQ5 iCycler Detection System (BioRad Laboratories, Ltd) was used for both protocols. A three-step PCR protocol (95°C for 10 min, followed by 40 cycles of 95°C for 15 sec, 60°C for 30 sec and 72°C for 30 sec) with SYBR-Green-based primers (sequences available upon request) and a two-step PCR protocol (95°C for 10 min, followed by 40 cycles of 95°C for 15 sec. and 60°C for 1 min), with FAM fluorophore was used for the TaqMan gene expression assay (ordered online from https://products.appliedbiosystems.com). The TaqMan gene expression assay ID for different targets were: GAPDH (Assay ID: Hs99999905_ml and VEGF-A (Assay ID: Hs00173626_ml. Relative quantification of mRNA was performed using the standard curve method using GAPDH as endogenous control. PCR efficiencies >90% were accepted. Analytical sensitivity performance characteristics were determined using ten-fold serial dilutions of pooled cDNA from twenty cases and controls as standard curve, and GAPDH as an internal endogenous control for the housekeeping gene. Intra-run replicates of serially diluted cDNA over the linear range of the assay demonstrated minimal variation in cycle threshold (Ct) values with a variation of <0.2 Ct, approximately corresponding to <5% variability in absolute levels.

### Flow cytometry

PBMC were isolated (as described above) from freshly collected blood samples obtained during clinical follow-up from five treatment naïve RRMS patients (2 females, 3 males; mean age 35) and nine RRMS patients treated with 2–24 infusions of natalizumab (4 females, 5 males; mean age 35 years). Cell populations sorted from PBMC included pure CD3^+^CD4^+^ (helper T cells), CD3^+^CD4^−^ (mainly cytotoxic T cells), CD19^+^ (B cells) and CD14^+^ (monocytes) in the treatment naïve MS patient group and CD3^+^CD4^+^ (helper T cells), CD3^+^CD8^+^ (cytotoxic T cells), CD3^+^CD4^+^CD25^high^ (regulatory T cells; Treg), CD3^−^C304^+^ (plasmacytoid dendritic cells; pDC) and monocyte gated cell populations in the natalizumab treated group. Cell populations were sorted from PBMC on a MoFlo high-speed cell sorter (DakoCytomation, Glostrup, Denmark) after staining with fluorescently labeled Abs from BD Biosciences, (San Jose, CA) and Miltenyi Biotec GmbH (Surrey, UK). After centrifugation, the cell pellets were lysed and prepared for RNA preparation and cDNA synthesis, with subsequent real-time PCR analysis using the SYBR-Green based protocol as described above.

### Genetic association study

A genetic association study was performed on 1114 Swedish patients with a MS diagnosis according to either Poser [27] or McDonald [24] (73% females, 27% males; mean age at sampling 53 years, IQR 22–91 years). The control group consisted of 1234 healthy blood donors (63% female and 37% male; mean age at sampling 47 years, IQR 21–76 years). Blood was sampled between 1987 and 2004 and genomic DNA was extracted from whole blood using standard procedures. Genotyping was performed with TaqMan SNP genotyping assay (Applied Biosystems) according to the manufacturer's instructions with 225 nM primer, 50 nM probe and 1 ng/µl genomic DNA in a total reaction volume of 5 µl. Further details are available upon request. Six SNPs tagging haplotype blocks across the entire VEGF-A gene on chromosome 6p21.3 were selected with Haploview using Tagger (r^2^ threshold = 0,8, LOD threshold for multi marker test = 3), (Table 1).

**Table 1:** Association analysis for risk of MS and single SNPs and haplotypes in *VEGF-A*.

SNP	Position	Genotype frequencies		Alleles	MAF		% GT		HWE P-value			P-value
		MM/Mm/mm^f^										Association
Rs #	Mb	Controls	MS	major∶minor	Hap	This	All	MS	All	Control	Case	Codominant
					Map	study						model
Rs833060^a^	43,843,127	712/424/71	616/337/71	G∶T	70.0	76.6	96.5	91.3	0.0264	0.4704	0.0112	0.3894
Rs699947^b^	43,844,367	333/598/311	274/493/255	A∶C	47.8^c^	51.0	98.3	91.2	0.0740	0.1922	0.2606	0.9986
Rs1570360^d^	43,845,808	570/495/125	413/375/110	G∶A	-e	67.6	91.4	80.1	0.0089	0.2525	0.0837	0.4114
Rs25648	43,846,955	567/280/25	682/290/28	C∶T	82.3	82.0	82.3	89.2	0.2453	0.1864	0.7407	0.3346
Rs3025030	43,858,565	866/336/28	734/269/32	G∶C	84.5	84.1	98.4	92.3	0.6415	0.5941	0.2486	0.4075
Rs10434	43,861,190	342/612/278	279/511/249	G∶A	56.2	52.1	98.0	92.7	0.7083	0.9090	0.6196	0.7142

No genetic association was found between SNPs in the VEGF-A gene and overall risk for MS in 1114 MS (n = 1114) healthy controls (n = 1234).

a VEGF-A exon 1 starts at position *43,845,931*. All positions according to NCBI SNP database.

b Also denoted −2578C/A. The AA genotype has lower VEGF-A levels [45], [46] and is associated with increased risk of amyotrophic lateral sclerosis in males [17] and increased risk of Alzheimer's disease [18].

c C is the major allele in HapMap. The frequency given in the table is for allele A.

d Also denoted −1154G/A. A-allele and AA-genotype lowers VEGF-A levels [45], [46].

e No allele frequency data available in HapMap or dbSNP.

f M = Major, m = minor allel.

### Statistical analysis

VEGF-A mRNA expression data in MS and controls were analyzed with the non-parametric Kruskal-Wallis test. For analysis of correlation between gender and VEGF-A level, the two-way ANOVA test was used with Bonferroni's post test. Correlations between VEGF-A levels and quantitative clinical measures (years of MS disease duration, EDSS, MSSS), age and correlations between VEGF-A in CSF mononuclear cells and PBMCs were analyzed with Spearman's rank test and non-linear regression for curve fit and the Kruskal-Wallis' test for multiple comparisons. The Mann-Whitney test for two-group comparison was used to compare VEGF-A levels between groups with either <9/≥9 MRI lesions, presence/absence of oligoclonal bands and normal/increased IgG-index. Analyses were carried out in GraphPad Prism 3.0 (GraphPad, San Diego, CA). Association analysis for risk of MS and single SNPs and haplotypes in *VEGF-A* was performed with R 2.8.0 using the package SNPassoc [28] and with Haploview 4.0. Power analysis was performed using genetic Power Calculator with a estimated prevalence for MS of 0.001 and a multiplicative model and type 1 error = 0.05 for allele test [29].

## Results

### VEGF-A mRNA expression in PBMC and CSF cells

**Figure 1:**
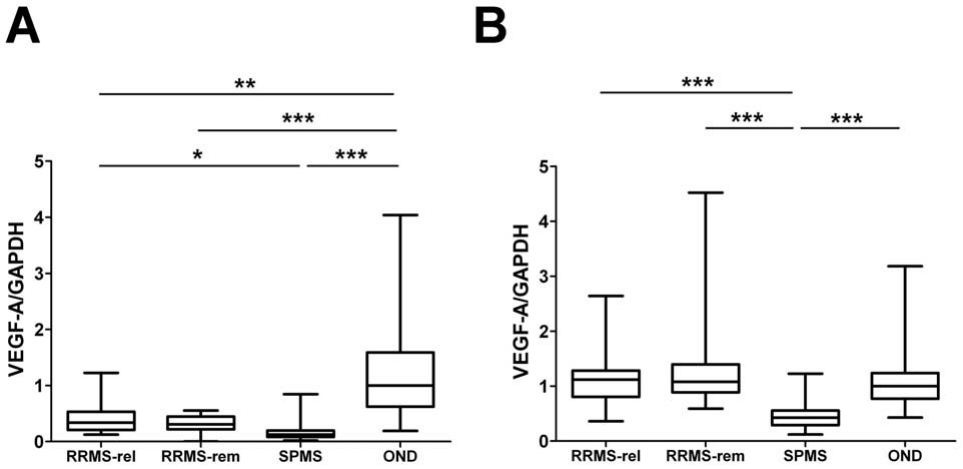
Box-whisker plot of VEGF-A mRNA expression in CSF cells and peripheral blood mononuclear cells (PBMC). Relative VEGF-A mRNA expression is reduced in CSF cells (A) in RRMS (n = 34) and SPMS (n = 32) compared to controls (OND; n = 47), and in PBMC (B) from SPMS (n = 35) compared to RRMS (n = 63) and OND (n = 68). No significant difference between RRMS in relapse and remission is seen in CSF cells (relapse n = 17, remission, n = 17) or PBMC (relapse n = 31, remission n = 32). *p<0.05; **p<0.01; ***p<0.001. All values were normalized to the median OND value in CSF cells and PBMC, respectively.

The relative expression of VEGF-A mRNA in CSF cells was drastically reduced both in RRMS and SPMS compared to controls as determined by SYBR-Green quantitative PCR in a first set of patients (*study group A*). Thus, RRMS displayed a three-fold and SPMS an eight-fold reduction of VEGF-A mRNA expression compared to OND controls in CSF cells (Figure 1A). In contrast, VEGF-A mRNA levels in PBMC were similar in RRMS and controls, but significantly reduced in SPMS (Figure 1B). No significant difference between RRMS in relapse and remission was found in CSF cells or PBMC. Both the findings of a down regulation of VEGF-A expression in CSF cells irrespective of disease course and the down regulation in PBMC solely in SPMS were corroborated in a separate set of patients (*study group B*) using a probe-based TaqMan PCR protocol (Figure S1A, B). In addition, there was a trend towards higher levels of VEGF-A expression in PBMC from RRMS sampled during a relapse as compared to patients in remission (Figure S1B).

Expression of VEGF-A in CSF cells or PBMC was not correlated to any measures of disease severity (EDSS, MSSS, disease duration), nor did the expression differ between groups with or without more than nine T2 MRI lesions, or oligoclonal bands and/or increased IgG index (data not shown). The expression level of VEGF-A mRNA in CSF cells and PBMC was positively correlated in the SPMS group in study group A (R^2^ = 0,17, p-value: 0.01612) and with borderline significance in study group B (R^2^ = 0,19, p-value 0,059). No significant correlation between VEGF-A mRNA expression in CSF cells and PBMC was observed in RRMS or in the control groups. In addition, neither gender nor age correlated with VEGF-A expression in PBMC or CSF cells (Figure S2).

### Monocytes as the main cellular source of VEGF-A mRNA

**Figure 2:**
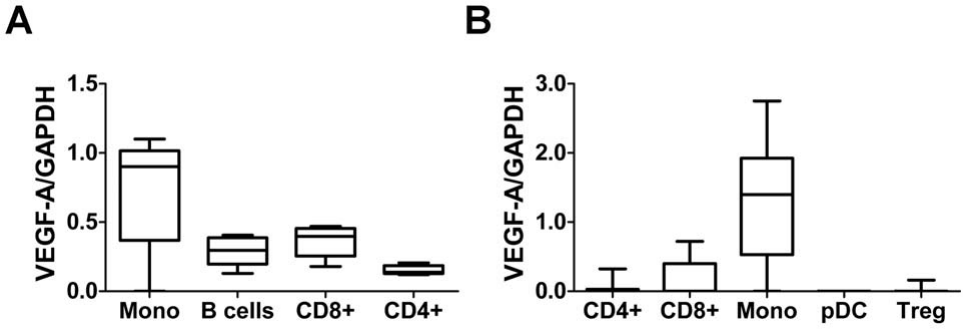
Box-whisker plot of VEGF-A mRNA expression in sorted peripheral blood mononuclear cell (PBMC) populations. Relative VEGF-A mRNA expression in sorted PBMC populations from (A) untreated RRMS (n = 5) and (B) RRMS treated with natalizumab (n = 9) demonstrate the highest expression of VEGF-A in the monocyte population in both groups. Monocytes were defined either as CD14^+^ (A) or by gating of forward and side scatter (B). pDC = plasmacytoid CD3^−^CD304^+^ cells, Treg = CD3^+^CD4^+^CD25^+^ cells.

In sorted PBMC populations from treatment naïve MS patients, expression of VEGF-A mRNA was found mainly in CD14^+^ monocytes (Figure 2A). In a prior study we detected expression of VEGF-A in mononuclear cell infiltrates in rats with experimental autoimmune encephalomyelitis (EAE) [22], suggesting that VEGF-A could potentially be expressed in T regulatory cells and/or myelin-specific autoreactive T cells that are enriched in the CNS of MS patients. We therefore took advantage of the therapeutic effect of natalizumab, which blocks the transmigration of lymphocytes across the blood-brain barrier resulting in peripheral sequestration of encephalitogenic T cells [30]. VEGF-A mRNA levels were determined in sorted PBMC from nine patients treated with natalizumab. Again, levels of VEGF-A were highest in monocytes and low or undetectable in the other analyzed cell populations (Figure 2B). Thus, in PBMC, monocytes constitute the major source of expression of VEGF-A, with some expression also in B cells and CD8^+^ T cells.

In order to exclude that reduced VEGF-A expression levels in PBMC from SPMS patients could be explained by difference in cellular composition, absolute and relative peripheral blood monocyte numbers were determined as part of a complete peripheral blood count in RRMS (n = 53), SPMS (n = 19) and controls (n = 58). This analysis did not reveal any discernible differences in the absolute or relative number of peripheral blood monocytes between the different groups (Table 2).

**Table 2:** Absolute and relative number of peripheral blood (PB) monocytes.

	RRMS	SPMS	OND	p-value
Absolute no. of PB monocytes,	0,4	0,4	0,3	ns
median (IQR) 10^9^/L	(0,3–0,5)	(0,3–0,4)	(0,3–0,4)	
Relative no. of PB monocytes,	0,25	0,24	0,21	ns
median (IQR) 10^9^/L	(0,17–0,3)	(0,18–0,53)	(0,40–0,25)	

No discernible difference in the absolute or relative number of peripheral blood monocytes, as determined by peripheral blood count, are evident between RRMS, SPMS and OND.

### Effect of natalizumab on VEGF-A mRNA expression

**Figure 3:**
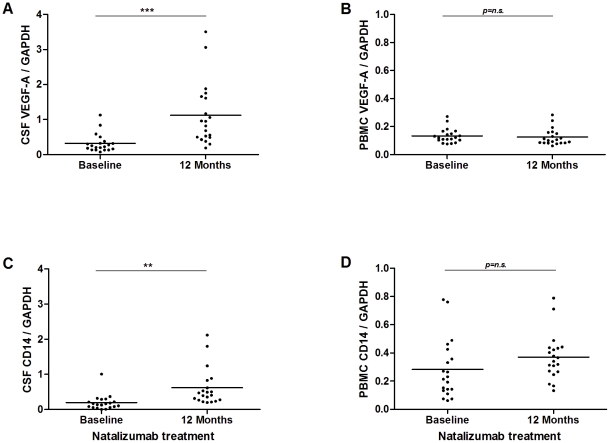
Box-whisker plot demonstrating the VEGF-A and CD14 mRNA expression in RRMS before and after natalizumab treatment. The effect of treatment on expression of VEGF-A in PBMC and CSF cells was studied in 19 RRMS patients prior to initiation of natalizumab and again after 12 months of treatment. Expression of CD14 was used as a marker of monocytes. There is a robust increase in CSF cell VEGF-A mRNA expression, (A) and a more moderate increase of CD14, (C) mRNA expression in CSF cells sampled after initiation of natalizumab treatment. In contrast, expression of VEGF-A and CD14 is not changed in PBMC after treatment with natalizumab (B, D). *p<0.05; **p<0.01; ***p<0.001.

In order to test if immunomodulatory treatment could alter the expression of VEGF-A, we studied the effect of natalizumab treatment in a set of RRMS patients. As monocytes express the highest levels of VEGF-A, we also estimated the relative proportion of monocytes by measuring the expression of the monocyte cell marker CD14. VEGF-A and CD14 mRNA in CSF cells and PBMC were determined in 19 RRMS patients (10 females, 9 males, mean age 40 years, IQR 33–49 years) prior to initiation of natalizumab and again after 12 months of treatment. There was a robust increase in CSF cell VEGF-A expression and a more moderate increase of CD14 mRNA expression in CSF cells, but no change in PBMC (Figure 3), suggesting both an effect on cellular CSF composition and a relative increased in VEGF-A expression.

### *VEGF-A* gene variants were not associated with risk of MS

The *VEGF-A* gene was genotyped with six SNPs in 1114 MS patients and 1234 controls covering haplotype blocks across the gene and promoter sequence. This study had 80% power to detect an odds ratio of 1.5 in the allelic test. No significant association between single SNP alleles or genotypes or *VEGF-A* haplotypes and overall risk for MS was found (Table 1). There were no gender differences in association of genotypes to MS. Furthermore, there was no correlation between *VEGF-A* alleles and VEGF-A expression in PBMC in a subset of MS patients (n = 85) and OND controls (n = 48). The sample size and the lack of detailed and updated phenotypic information on disease course excluded the possibility to perform association to risk of progressive MS.

## Discussion

We here reproduce, in a large cohort, our previous findings that VEGF-A mRNA expression is reduced in CSF cells of MS patients [22]. However, the main novel finding of the present study is that SPMS patients are characterized by a prominent decrease of VEGF-A mRNA expression in PBMC compared to both RRMS and controls, who display comparable levels of VEGF-A expression. We could not demonstrate any significant correlation to age or a number of clinical or paraclinical parameters, such as EDSS, MSSS, disease duration or number of T2 MRI lesions, which is in agreement with a previous study [21].These results suggest that down regulation of VEGF-A mRNA expression in PBMC reflects an underlying disease mechanisms that operates specifically in progressive MS. This finding is of interest in the context of emerging evidence that connects dysregulation of VEGF-A to neurodegenerative processes [31]. Thus, VEGF-A protects neurons from environmental stress and apoptosis and can directly stimulate neurogenesis and axonal regeneration [10], [31]. Targeting of the hypoxia-response element in the *VEGF* gene leads to degeneration of motorneurons and intracerebroventricular delivery of VEGF-A in a rat model of ALS delays onset of motorneuron degeneration and prolongs survival [12], [14]. The sensitivity of motorneurons to dysregulation of VEGF-A is interesting in light of the recent demonstration of a major loss of spinal motorneurons also in MS [32]. Of note, Seabrook *et al* demonstrated decreased levels of VEGF-A protein in rat spinal cord tissue during EAE relapse and a reduced expression in neurons upon immunohistochemistry [33]. Other studies on RRMS have reported increased VEGF-A mRNA expression in smaller groups of MS patients during relapse compared to remission and/or controls in peripheral blood and CSF [21], [23]. In addition, Su *et al* showed that the higher serum levels of VEGF-A decreased within one month from relapse onset and tended to be lower than healthy controls during remission. An increase of VEGF-A in the initial phases of relapse is compatible with its role as a pro-inflammatory factor that attracts monocytes and lymphocytes, upregulates immunomodulatory adhesion molecules, stimulates secretion of proinflammatory cytokines, and increases blood-brain barrier permeability [22], [34]. We observed a tendency towards higher VEGF-A mRNA levels in relapse compared to remission only in one of the studied cohorts. This discrepancy may be related to the timing of sampling in relation to onset of relapse symptoms or the occurrence of subclinical disease activity in the remission group. It is known that the net effect of VEGF-A can vary depending on the target tissue, the timing and the concentration [35]. Neuroprotection of the ischemic brain is critically dependent on proper dosage and may be compromised by angiogenesis, which may explain why VEGF-A in some studies show increased expression [19], [21], [36] and correlate to exacerbation of disease in EAE [19], [37], while others report decreased expression [22], [38]. We measured VEGF-A protein levels with ELISA in a small subset of patients for which plasma samples were available, revealing a non significant trend towards decreased levels in SPMS patients compared to RRMS and controls (data not included). Replication in larger materials is therefore needed to demonstrate if VEGF-A expression differences also are translated into changes in protein levels in peripheral blood.

The neurodegenerative disorders ALS and AD disease have been associated with a genetic variation in the *VEGF-A* gene (−2578AA; rs699947) associated with reduced VEGF-A levels [17], [18]. We were here unable to demonstrate any haplotype dependent association to overall risk of MS. However, the genetic contribution of the described SNP is modest, since meta-analyses suggest an overall 1.14–1.19 fold increased risk of ALS or AD in subjects carrying the −2578AA genotype [17], [18] and relative risks of this magnitude are too small to be detected by our study. Larger and better characterized clinical materials are therefore needed to rule out a genetic association between *VEGF-A* and MS in general or to SPMS in particular.

The observation that monocytes are the main cellular source of VEGF-A expression raises the question if differences in the relative proportion of monocytes could affect results obtained from mixed cell populations. However, this was not the case in the periphery, since absolute and relative monocyte numbers were similar across the groups, in agreement with a previous study [39]. In contrast, the finding of reduced VEGF-A expression in CSF mononuclear cells at least in part may be explained by a relative decrease of monocyte numbers in the CSF of MS patients compared to controls [40]. We also studied the effect of natalizumab on VEGF-A expression, demonstrating a robust increase in expression by this therapy. This may be a result of a relative increase in CSF monocyte numbers, but likely also reflects a direct or indirect pharmacological effect of natalizumab on the phenotype of CSF monocytes since the treatment-induced effect on expression of CD14 was smaller.

The finding of an altered VEGF-A expression profile connected to monocytes is interesting since accumulating evidence supports a role for innate immune mechanisms in progressive MS. Thus, dendritic cells of SPMS patients secrete more proinflammatory cytokines and increased monocyte CD86 expression and IL-12 secretion is associated with transition to a progressive MS phase [41], [42]. Taken together this suggests that the transition to a progressive disease course is connected with a change in underlying disease mechanisms. Indeed, the observations that clinical deterioration in SPMS generally occurs in the absence of novel inflammatory lesions and that it does not respond to traditional forms of immunomodulatory treatment support the notion that other disease mechanisms are operative [43], [44].

In conclusion, we here demonstrate that expression of VEGF-A is down regulated in CSF cells irrespective of disease course, but that solely SPMS patients display down regulated VEGF-A expression in PBMC as compared to both RRMS and controls. These results extend prior observations on peripheral innate immune changes in SPMS and point towards a possible target for future disease modulatory drugs. Also, this finding may prove to be useful as a biomarker for SPMS, providing support for patient management and decisions on therapeutic interventions. Further studies are needed to address the question if dysregulated VEGF-A expression in MS is a cause or consequence of a progressive disease phenotype.

## Supporting Information

Figure S1 **Confirmatory analysis of VEGF-A mRNA expression in CSF cells and PBMC in a separate set of patients (** ***study group B*** **) composed of RRMS (n = 65), SPMS (n = 20) and controls (OND; n = 48).** Expression of VEGF-A mRNA is decreased in CSF cells in RRMS and SPMS compared to OND (A). As in study group A, expression of VEGF-A mRNA is decreased in SPMS compared to both RRMS and controls (B). A non-significant trend towards higher expression of VEGF-A in PBMC is evident in RRMS sampled during a relapse (n = 14) compared to remission (n = 51). No significant differences between RRMS in relapse (n = 14) and remission (n = 51) was found in CSF cells. *p<0.05; **p<0.01; ***p<0.001. All values were normalized to the median OND value in CSF cells or PBMC.
(TIF)

Figure S2 **Age span in the analyzed MS and control groups (A).** Patients with SPMS were older than both RRMS patients and controls with OND. No significant correlation between VEGF-A levels in CSF and age is detected in RRMS, SPMS or the pooled MS group (B). In PBMC age is not correlated to VEGF-A levels in the RRMS and SPMS groups. However, a weak correlation is evident in the pooled MS group, mostly reflecting the lower levels of VEGF-A in SPMS patients (C). Correlation was analyzed with Spearman's rank test and line fit was determined with non-linear regression.
(TIF)

Table S1 **Characteristics of patients and samples included in the expression analysis.**
(DOC)

Table S2 **Diagnoses of the patients included in the control group (OND).**
(DOC)
