# Supplementary figures and images for: The Expression of VEGF-A Is Down Regulated in Peripheral Blood Mononuclear Cells of Patients with Secondary Progressive Multiple Sclerosis
Source: PLoS One. 2011 May 6;6(5):e19138. doi: 10.1371/journal.pone.0019138 (PMC3089609; doi:10.1371/journal.pone.0019138)

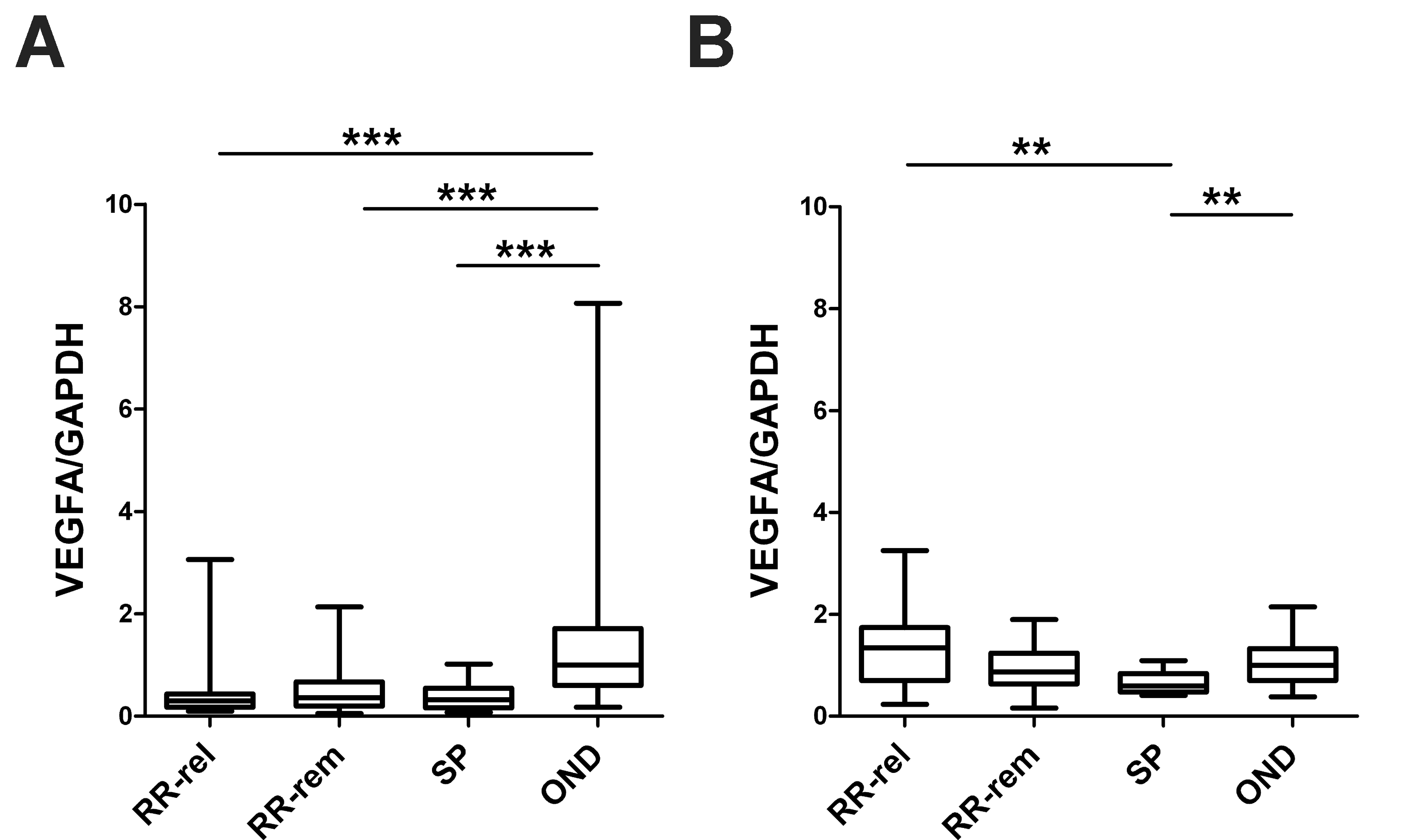

Supplement: Figure S1 — Confirmatory analysis of VEGF-A mRNA expression in CSF cells and PBMC in a separate set of patients ( study group B ) composed of RRMS (n = 65), SPMS (n = 20) and controls (OND; n = 48). Expression of VEGF-A mRNA is decreased in CSF cells in RRMS and SPMS compared to OND (A). As in study group A, expression of VEGF-A mRNA is decreased in SPMS compared to both RRMS and controls (B). A non-significant trend towards higher expression of VEGF-A in PBMC is evident in RRMS sampled during a relapse (n = 14) compared to remission (n = 51). No significant differences between RRMS in relapse (n = 14) and remission (n = 51) was found in CSF cells. *p<0.05; **p<0.01; ***p<0.001. All values were normalized to the median OND value in CSF cells or PBMC. (TIF) [file pone.0019138.s001.tif]

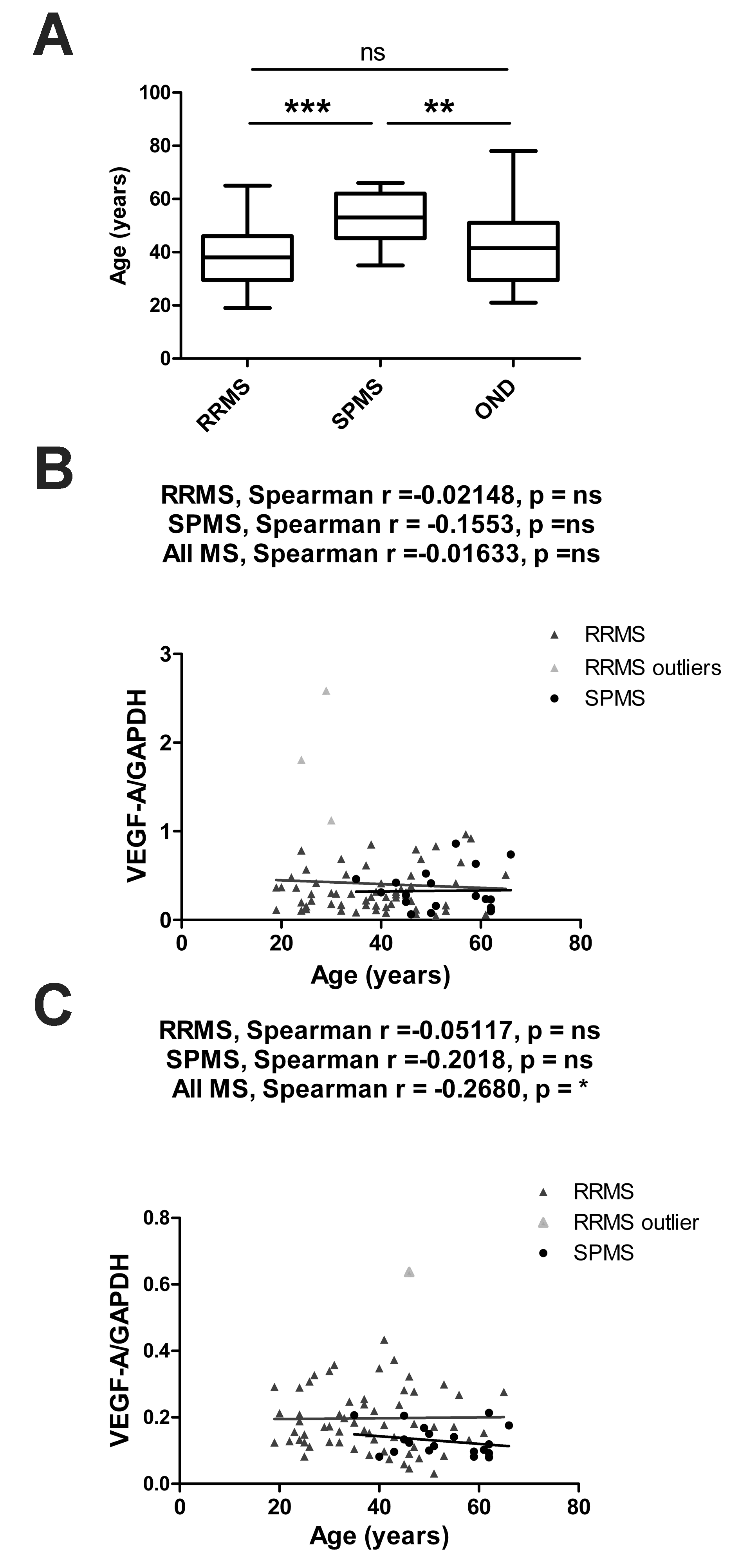

Supplement: Figure S2 — Age span in the analyzed MS and control groups (A). Patients with SPMS were older than both RRMS patients and controls with OND. No significant correlation between VEGF-A levels in CSF and age is detected in RRMS, SPMS or the pooled MS group (B). In PBMC age is not correlated to VEGF-A levels in the RRMS and SPMS groups. However, a weak correlation is evident in the pooled MS group, mostly reflecting the lower levels of VEGF-A in SPMS patients (C). Correlation was analyzed with Spearman's rank test and line fit was determined with non-linear regression. (TIF) [file pone.0019138.s002.tif]
