# Supplementary material for: The Expression of VEGF-A Is Down Regulated in Peripheral Blood Mononuclear Cells of Patients with Secondary Progressive Multiple Sclerosis
Source: PLoS One. 2011 May 6;6(5):e19138. doi: 10.1371/journal.pone.0019138 (PMC3089609; doi:10.1371/journal.pone.0019138)
Supplement: Table S1 — Characteristics of patients and samples included in the expression analysis. (DOC) [file pone.0019138.s003.doc]

Table S1. Characteristics of the patients and samples included in the expression analysis.

| **Study group A** | **RRMS** | **SPMS** | **OND** |
| --- | --- | --- | --- |
| No. patients | 63 | 35 | 68 |
| Female/Male | 38/25 | 22/13 | 52/16 |
| Age, median (IQR), years | 35 (29-42) | 55 (48-60) | 38 (32-49) |
| EDSS, median (IQR) | 2 (2-3,5) | 5,5 (5-6) | n.a |
| Disease duration, median (IQR) years | 3 (2-7) | 24 (19-35) | n.a |
| Samled during relapse/remission | 31/32 | 0% | n.a |
| % OCB positive | 90% | 80%^a^ | 0% |
| CSF mononuclear cells/μL, median (IQR) | 7 (4-13) | 3 (2-5)^a^ | 2 (1-2) |
| CSF albumin mg/ml, median (IQR) | 229 (177-290) | 263 (196-356)* | 195 (150-243) |
| PBMC samples analyzed, no. | 63 | 35 | 68 |
| CSF samples analyzed, no. | 34 | 32 | 47 |
| Paired PBMC and CSF samples analyzed, no. | 34 | 32 | 47 |
| **Sudy group B** |  |  |  |
| No. patients | 65 | 20 | 48 |
| Female/Male | 43/22 | 10/10 | 35/13 |
| Age, median (IQR), years | 38 (30-46) | 53 (46-62) | 42 (30-50) |
| EDSS, median (IQR) | 2 (1-4) | 5,5 (4,5-6) | n.a |
| Disease duration, median (IQR), years  median ( IQR) | 5 (2-9) | 13 (8-19) | n.a |
| Samled during relapse/remission | 14/ 51 | 0% | n.a |
| % OCB positive | 89% | 100% | 0% |
| CSF mononuclear cells/ μL, median (IQR)  mmmm mmmmmmmmedian median (IQR) | 5 (2-9) | 3 (1-5) | 1 (1-3) |
| CSF albumin mg/ml, median (IQR) | 219 (189-257) | 222 (193-365) | 263 (172-267) |
| PBMC samples analyzed, no. | 65 | 20 | 48 |
| CSF samples analyzed, no. | 65 | 20 | 48 |
| Paired PBMC and CSF samples analyzed, no. | 65 | 20 | 48 |

a: 23 patients tested
