# Supplementary material for: The Expression of VEGF-A Is Down Regulated in Peripheral Blood Mononuclear Cells of Patients with Secondary Progressive Multiple Sclerosis
Source: PLoS One. 2011 May 6;6(5):e19138. doi: 10.1371/journal.pone.0019138 (PMC3089609; doi:10.1371/journal.pone.0019138)
Supplement: Table S2 — Diagnoses of the patients included in the control group (OND). (DOC) [file pone.0019138.s004.doc]

Table S2. Diagnosis of the patients in the control group (OND).

| **Study group A (n=68)** | **Study group B (n=48)** |
| --- | --- |
| Atypical facial pain (n=1)  B12-deficiency with sensory symptoms (n=1)  Carpal tunnel syndrome (n=2)  Cerebral metastasis (n=1)  Cerebrovascular disease (n=4)  Cervical meningeoma (n=1)  Cervicobrachialgia (n=1)  Idiopathic intracranial hypertension (n=5)  Ischemic opticus neuritis (n=1)  CNS lymphomatoid granulomatosis (n=1)  Migraine (n=3)  Monoparesis (n=1)  Neuralgia (n=4)  Neurastenia (n=1)  Painsyndrome (n=1)  Polyneuropathy (n=2)  Postcommotio syndrome (n=1)  Pudendal nerve damage (n=1)  Lumbar spine sarcoma (n=1)  Sensory symptoms (n=27)  Shoulder neuritis (n=1)  Unspecific white matter lesions (n=6)  Vertigo (n=1) | Autonomic neuropathy (n=1)  Cervical ependymoma (n=1)  Hereditary spastic paresis (n=1)  Cerebrovascular disease (n=1)  Migraine (n=1)  Neurastenia (n=2)  Neuromuscular bladder disturbance (n=2)  Neuropathy (n=2)  Parkinsons disease (n=1)  Psychosis (n=10)  Rhizopathy (n=1)  Sensory symptoms (n=11)  Spinal stenosis (n=2)  Spondylosis (n=1)  Tension headache (n=3)  Neuralgia (n=1)  Unspecific white matter lesions (n=1)  Vertebral dissection (n=1)  Vertigo (n=5) |
